# Species diversity of tick and tick-borne pathogens from roe deer (*Capreolus pygargus tianschanicus*), including new record of *Haemaphysalis megaspinosa* (Acari: Ixodidae), in Jeju Island, Republic of Korea

**DOI:** 10.1016/j.ijppaw.2025.101125

**Published:** 2025-07-31

**Authors:** Jong-Uk Jeong, Hyun-Jeong Kim, Da-Seul Seong, Hae-Eun Kang, Jeong-Hee Han, Kwang Shik Choi, In-Soon Roh

**Affiliations:** a Vector-Borne Disease Laboratory, Foreign Animal Disease Division, Animal and Plant Quarantine Agency, Gimcheon, 39660, Republic of Korea; b Laboratory Animal Research Center, Central Scientific Instrumentation Facility, Gyeongsang National University, Jinju, 52828, Republic of Korea; c College of Veterinary Medicine, Kangwon National University, Chuncheon, 24341, Republic of Korea; d Department of Biology, College of Natural Sciences, Kyungpook National University, Daegu, 41566, Republic of Korea

**Keywords:** Hard ticks, Roe deer, Tick-borne pathogens, Jeju island, Republic of Korea

## Abstract

Hard ticks (Ixodidae family) are primary vectors of zoonotic diseases, including severe fever with thrombocytopenia syndrome (SFTSV), anaplasmosis, babesiosis, Lyme borreliosis, and ehrlichiosis. The roe deer (*Capreolus pygargus tianschanicus*), a key host for tick-borne diseases, is widely distributed on Jeju Island, Republic of Korea (ROK). Although the increased interactions between deer, livestock, and humans have raised concerns about zoonotic disease transmission, this area has remained understudied. Therefore, this study aimed to investigate the species diversity of ticks infesting roe deer and the prevalence of major tick-borne diseases in this region. From 2018 to 2020, 1832 ticks were collected from 154 roe deer. Four tick species were identified: *Haemaphysalis longicornis* (50.1 %), *H*. *flava* (47.9 %), *Ixodes nipponensis* (1.7 %), and *H*. *megaspinosa* (0.3 %). This study reports the first detection of *H. megaspinosa* in the ROK. Pathogen screening detected *Anaplasma* spp. (minimum infection rate, MIR: 0.38 %), *Babesia* spp. (0.05 %), and *Ehrlichia* spp. (0.44 %); however, sequencing was only successful for *Anaplasma* spp. These findings highlight the importance of continued tick surveillance and research on the zoonotic risks associated with emerging tick species in the ROK.

## Highlights

• Roe deer on Jeju Island, ROK may act as reservoirs for tick-borne diseases (TBDs).
• We identified four tick species infesting roe deer.
• *Haemaphysalis megaspinosa* was identified for the first time in the ROK.
• Continued surveillance is crucial for detecting emerging tick species in Korea.
• Our results may guide future strategies to mitigate TBD risks.

## Graphical abstract

**Figure:**
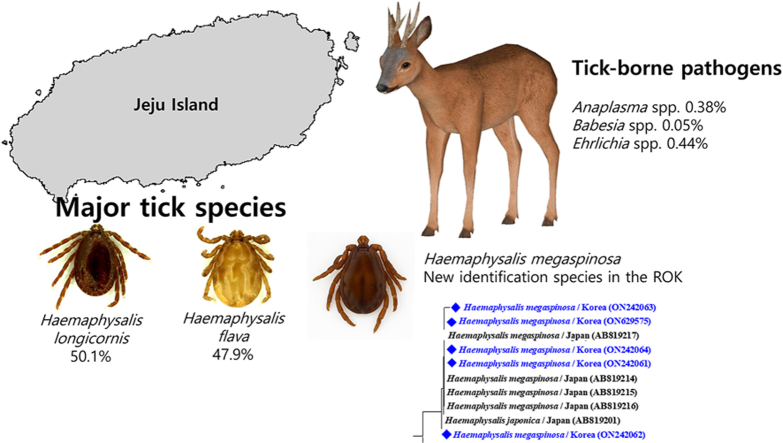

## 1 Introduction

Ticks are obligate hematophagous ectoparasites that act as vectors for various pathogens, including viruses, bacteria, and protozoa, posing significant risks to human and animal health (Inokuma et al., 2002; Li et al., 2022; Zhang et al., 2017). The incidence of tick-borne diseases is increasing owing to climate change, habitat expansion, and increased human-wildlife interactions (Im et al., 2019; Korea Disease Control and Prevention Agency, 2024; Lee and Chung, 2023).

In several European studies, roe deer (*Capreolus capreolus*) have been shown to harbor a variety of tick-borne pathogens such as *Babesia*, *Ehrlichia*, and tick-borne encephalitis virus, acting as both potential reservoir hosts and effective sentinel species for monitoring zoonotic disease circulation (Gerth et al., 1995; Liz et al., 2002; Duh et al., 2005).

In the Republic of Korea (ROK), the related species *C. pygargus tianschanicus* is distributed nationwide but is particularly abundant on Jeju Island, where it is frequently observed in forested, agricultural, and peri-urban environments. This high density, along with their ecological overlap with domestic animals and humans, suggests that *C. p. tianschanicus* may likewise serve as a suitable sentinel species for regional tick-borne pathogen surveillance. Several ecological traits support their effectiveness as sentinels. They have regular exposure to tick vectors and often share habitats with livestock and humans. Additionally, their relatively high population density and the feasibility of carcass-based sampling allow for consistent and long-term monitoring.

Supporting this possibility, recent studies in northeastern Asia, including China, have detected pathogens such as *Anaplasma* and *Borrelia* in wild *C. p. tianschanicus*, suggesting that this species may serve not only as a host for ticks but also as a reservoir for specific tick-borne pathogens (Byun et al., 2024; Wang et al., 2019).

Therefore, this study aimed to assess the species diversity of ticks infesting roe deer on Jeju Island and to evaluate infection rates for five major tick-borne pathogens: *Anaplasma*, *Babesia*, *Borrelia*, *Ehrlichia*, and severe fever with thrombocytopenia syndrome virus (SFTSV).

## 2 Materials and methods

### 2.1 Tick collection and identification

Between 2018 and 2020, 154 roe deer (*C*. *p*. *tianschanicus*) were legally hunted on Jeju Island, ROK (33.367°N, 126.533°E). Ticks were collected from each animal's fur using sterilized tweezers and individually stored in 1.5 mL microcentrifuge tubes. Samples were kept at −70 °C until further processing.

All collected ticks were morphologically identified to species level using a stereo microscope (Olympus SZ61, Japan) and the morphological keys of Yamaguti et al. (1971). Species, developmental stage, and sex were recorded. Ticks exhibiting morphological features distinct from known species were further analyzed.

### 2.2 Molecular identification

Partial 16S rRNA gene sequences were obtained from representative samples to confirm species identification. Each tick was incubated in 200 μL lysis buffer and 20 μL of proteinase K from the Maxwell RSC Viral Total Nucleic Acid Purification Kit (Promega, Wisconsin, USA) without prior homogenization. The mixture was incubated overnight at 56 °C. According to the manufacturer's instructions, nucleic acid was extracted using the Maxwell RSC system. PCR amplification targeted a 450 bp fragment of the 16S rRNA gene, as expected from agarose gel electrophoresis results following the protocol of Black and Piesman (1994). Amplified products were purified and sequenced by Bionics Co., Ltd. (Daejeon, ROK). Sequences were aligned using MEGA7 software and compared with GenBank entries via the BLAST algorithm.

### 2.3 Tick pooling

1832 ticks were grouped into 201 pools based on the following criteria: ticks collected from the same host, belonging to the same species, and sharing the same sex and developmental stage. Each pool contained up to 30 ticks and was subjected to downstream nucleic acid extraction and pathogen detection.

### 2.4 Tick-borne pathogens screening

Minimum infection rates (MIRs) were calculated as the number of positive pools divided by the total number of ticks tested, expressed as a percentage.

Tick-borne pathogen screening targeted five major pathogens: *Anaplasma* spp., *Babesia* spp., *Borrelia* spp., *Ehrlichia* spp., and severe fever with thrombocytopenia syndrome virus (SFTSV).

For pooled samples, nucleic acids were extracted using the Maxwell RSC Viral Total Nucleic Acid Purification Kit (Promega, Wisconsin, USA) following the manufacturer's protocol. Each pool was homogenized in 1 mL of cell culture medium consisting of α-MEM (500 mL) supplemented with 10 mL of Antibiotic-Antimycotic solution (100X; Gibco, Thermo Fisher Scientific) using Precellys homogenization tubes and a Precellys tissue homogenizer (Bertin Instruments, France). Extracted total nucleic acids (including both DNA and RNA) were used as templates for subsequent qPCR and conventional PCR assays.

Pathogen detection was conducted using AnyQvet Tick-borne Disease qPCR I and II kits (KoreaGentec, Chuncheon, ROK). Real-time qPCR protocols were performed according to the manufacturer's instructions. qPCR I targets the S and M segments of SFTSV and several Babesia species. qPCR II targets *Anaplasma* spp., *Borrelia* spp., and *Ehrlichia* spp., including multiple zoonotic strains.

PCR amplification for sequencing was performed on qPCR-positive samples using pathogen-specific primers adapted from Park et al. (2019). Expected amplicon sizes were approximately 420 bp for *Anaplasma* spp. and *Babesia* spp., and 340 bp for *Ehrlichia* spp., based on primer design and gel electrophoresis results. AccuPower PCR PreMix kits (Bioneer, Daejeon, ROK) were used under the following cycling conditions: 95 °C for 5 min; 35 cycles of 94 °C for 30 s, 56–60 °C for 30 s, 72 °C for 1 min; and a final extension at 72 °C for 10 min. Negative controls (nuclease-free water) were included in each qPCR and conventional PCR run to monitor potential contamination. Amplified products were visualized on 1.5 % agarose gels, purified, and sequenced by Bionics Co., Ltd.

### 2.5 Sequence and phylogenetic analysis

Obtained sequences were aligned using MEGA7 and analyzed for genetic variation (Kumar et al., 2016). Phylogenetic trees were constructed using the maximum likelihood method with reference sequences retrieved from GenBank.

## 3 Results

### 3.1 Tick species diversity

1832 ticks from four species were collected from 154 roe deer. The dominant tick species infesting roe deer on Jeju Island were *Haemaphysalis longicornis* (n = 918, 50.1 %; 291 females, 411 males, and 216 nymphs) and *H*. *flava* (n = 877, 47.9 %; 136 females, 678 males, and 63 nymphs), followed by *Ixodes nipponensis* (n = 32, 1.7 %; 18 females, 13 males, and one nymph) and *H. megaspinosa* (n = 5, 0.3 %; 3 females and two males) (n = 5, 0.3 %) (Table 1). This marks the first record of *H. megaspinosa* infesting roe deer in the ROK. On average, approximately 11.9 ticks were collected per deer.

**Table 1:** Ticks collected on roe deer in Jeju Island, Republic of Korea, from 2018 to 2020.

Year	Host	Number of hosts	*Haemaphysalisa longicornis*			*Haemaphysalisa flava*			*Haemaphysalisa magaspinosa*			*Ixodes nipponesis*		
			Female	Male	Nymph	Female	Male	Nymph	Female	Male	Nymph	Female	Male	Nymph
2018	Roe deer	8	9	6	1	41	151	12	0	1	0	8	3	0
2019	Roe deer	86	140	108	128	82	398	47	2	1	0	10	10	1
2020	Roe deer	60	142	294	87	13	129	4	1	0	0	0	0	0

Total		154	291	411	216	136	678	63	3	2	0	18	13	1

### 3.2 Morphological and molecular characteristics of *H. megaspinosa*

**Fig. 1:**
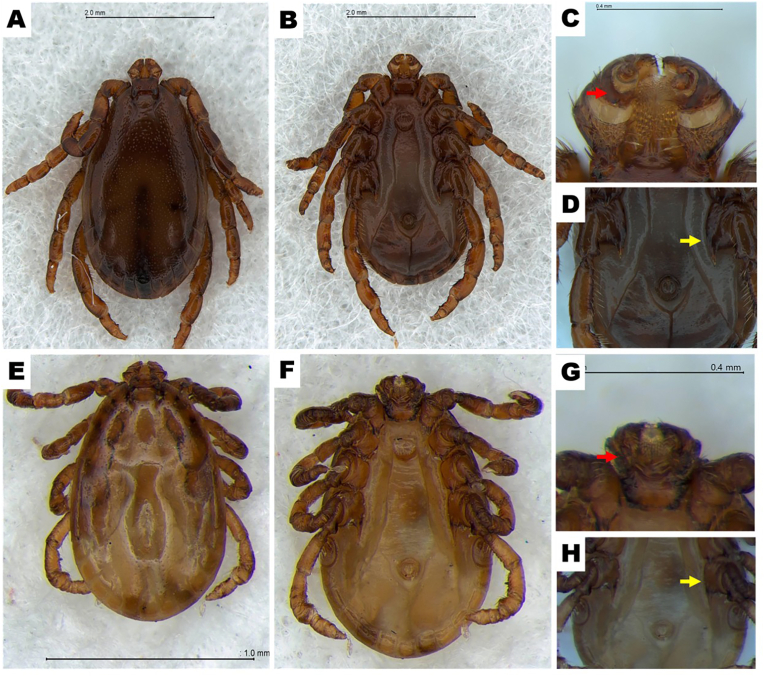
Morphological feature of male *Haemaphysalis megaspinosa* (A, B, C, and D) and male *H. flava* (E, F, G, and H). (A and E) dorsal view; (B and F) ventral view; (C and G) ventral view of the capitulum; (D and H) ventral view of coxa Ⅳ. C and G indicate posteroexternal juncture of palpal segment Ⅱ (red arrow). D and H indicate spur of coxa Ⅳ (yellow arrow).

The morphological analysis identified three female and two male *H. megaspinosa*, distinguishable from *H. flava* by their larger size and brownish coloration (Fig. 1A–H). In *H. megaspinosa,* the posteroexternal juncture of palpal segment II was short and blunt (Fig. 1C–G), while the coxa IV spur in males was short, wide, and curved, in contrast to the narrow, long, and straight spur of *H. flava* (Fig. 1D–H).

**Fig. 2:**
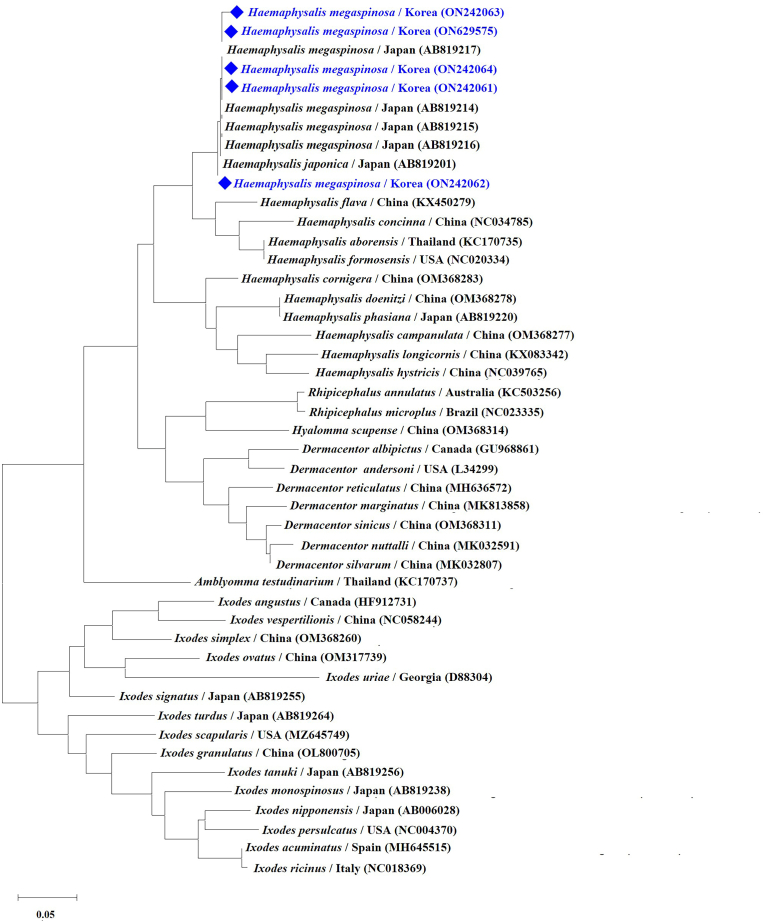
Phylogenetic tree based on the ticks 16S rRNA gene. The tree was constructed using the maximum likelihood method. Sequences obtained in this study were marked with solid diamond forms. Bar = 0.05 length.

Partial 16S rRNA sequences showed 99.25–100 % similarity to Japanese *H. megaspinosa* sequences in GenBank (Accession numbers ON242061–64 and ON629575). Sequence comparisons among *H. megaspinosa* samples collected in this study revealed minor genetic variations, with the ON242062 sequence exhibiting a single nucleotide polymorphism (SNP) and the ON242063 sequence displaying three SNPs. Phylogenetic analysis confirmed *H. megaspinosa* as a distinct clade, clearly separated from other tick species. However, *H. japonica* clustered closely with *H. megaspinosa* (Fig. 2).

### 3.3 Tick-borne pathogen screening

A total of 201 tick pools were screened for five Tick-borne disease pathogens. The MIR of *Anaplasma*, *Babesia,* and *Ehrlichia* were 0.38 % (seven pools/1832 ticks), 0.05 % (1/1832), and 0.44 % (8/1832), respectively. The MIR of *Anaplasma* spp. was 0.54 % (5/918) in *H. longicornis*, 0.11 % (1/877) in *H. flava*, and 3.13 % (1/32) in *I. nipponesis*. The MIR of *Babesia* spp. was 20.0 % (1/5) in *H. megaspinosa.* The MIR of *Ehrlichia* spp. was 0.11 % (1/918) in *H. longicornis* and 0.80 % (7/877) in *H. flava*. None of the samples tested positive for *Borrelia* spp. or SFTSV (Table 2).

**Table 2:** Detection of five pathogens from ticks (pools) collected on roe deer in Jeju Island, Republic of Korea.

	Number of tested ticks	Number of pools	*Anaplasma* spp.		*Babesia* spp.		*Borrelia* spp.		*Ehrlichia* spp.		SFTSV	
			Number of positive tick pools	MIR (%) of ticks	Number of positive tick pools	MIR (%) of ticks	Number of positive tick pools	MIR (%) of ticks	Number of positive tick pools	MIR (%) of ticks	Number of positive tick pools	MIR (%) of ticks
*Haemaphysalisa longicornis*	918	87	5	0.54 (5/918)	0	0 (0/918)	0	0 (0/918)	1	0.11 (1/918)	0	0 (0/918)
*Haemaphysalisa flava*	877	97	1	0.11 (1/877)	0	0 (0/877)	0	0 (0/877)	7	0.80 (7/877)	0	0 (0/877)
*Haemaphysalisa megaspinosa*	5	5	0	0 (0/5)	1	20.0 (1/5)	0	0 (0/5)	0	0 (0/5)	0	0 (0/5)
*Ixodes nipponesis*	32	12	1	3.13 (1/32)	0	0 (0/32)	0	0 (0/32)	0	0 (0/32)	0	0 (0/32)
Total	1832	201	7	0.38 (7/1832)	1	0.05 (1/1832)	0	0 (0/1832)	8	0.44 (8/1832)	0	0 (0/1832)

SFTSV, severe fever with thrombocytopenia syndrome; MIR, minimum infection rate (number of positive tick pools/total number of ticks tested ✕ 100).

### 3.4 Tick-borne pathogen identification

**Fig. 3:**
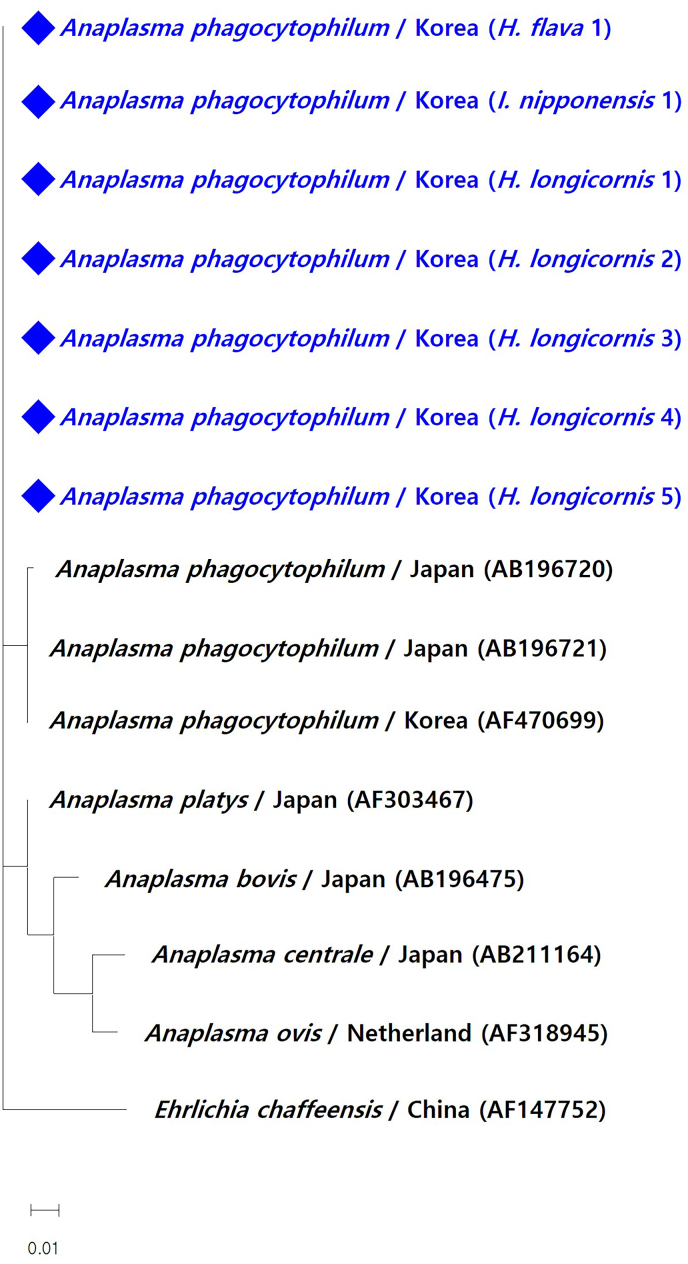
Phylogenetic tree based on the *Anaplasma* spp. and *Ehrlichia chaffeensis* 16S rRNA gene. The tree was constructed using the maximum likelihood method. Sequences obtained in this study were marked with solid diamond forms. Bar = 0.01 length.

Positive pools for *Anaplasma* spp. were successfully amplified for sequencing. The results revealed that all *Anaplasma*-positive samples were identified as *A. phagocytophilum*. Phylogenetic analysis, performed using the maximum likelihood method, included these sequences alongside reference sequences of *A. phagocytophilum*, other *Anaplasma* spp., and *E*. *chaffeensis* retrieved from GenBank (Fig. 3). The analysis revealed that the *A. phagocytophilum* sequences obtained in this study clustered with other *A. phagocytophilum* sequences, displaying minor variations. Moreover, they were distinct from other *Anaplasma* species, including *A. platys*, *A. bovis*, *A. centrale*, and *A. ovis*. Samples that tested positive for *Babesia* spp. and *Ehrlichia* spp. During the screening, the tests were subjected to PCR analysis; however, none of these samples were successfully amplified.

## 4 Discussion

Due to climate change, Jeju Island is transitioning from a temperate to a subtropical climate (Korea Meteorological Administration, 2024). Rising temperatures are expected to impact tick populations and the prevalence of tick-borne diseases, making Jeju an ideal location for studying vector ecology (Lee and Chung, 2023; Ribas-Deulofeu et al., 2023).

Roe deer, which have recently increased in numbers due to conservation efforts, frequently interact with humans and livestock, raising concerns about the transmission of zoonotic diseases (Baek and Lee, 2021; Lee et al., 2015). Previous studies on Jeju Island have consistently identified *H. longicornis* as the dominant tick species (Kim et al., 2011; Moon et al., 2009, 2014), and our results corroborate this finding.

While *H. megaspinosa* has not been previously reported in the ROK, we identified it morphologically and genetically as a novel record in this region. Five specimens were collected over a three-year period, all from roe deer on Jeju Island. The detection of *H. megaspinosa*, which showed close genetic similarity to Japanese isolates, suggests a possible recent introduction. Passive introduction via migratory birds is plausible, as has been observed for *H. concinna* in Jeju (Choi et al., 2014). However, given the limited sample size, this finding should be interpreted as preliminary. At present, there is insufficient evidence to assess the ecological distribution or epidemiological relevance of *H. megaspinosa* in ROK. This interpretation, consistent with earlier reports of bird-mediated tick dispersal, requires further investigation. *H. megaspinosa* has also been reported to parasitize humans in Japan (Seishima et al., 2000), suggesting potential zoonotic implications.

Regarding tick-borne pathogens, this study confirmed the presence of *Anaplasma* spp., *Babesia* spp., and *Ehrlichia* spp. across different tick species. *Anaplasma* spp. was detected in multiple species and successfully sequenced as *A. phagocytophilum*, a zoonotic agent associated with human granulocytic anaplasmosis. *A. phagocytophilum* was detected in various tick species, indicating its circulation among ticks infesting roe deer on Jeju Island.

The detection of *A*. *phagocytophilum* in multiple tick species collected from roe deer suggests that *C*. *pygargus* may play a role in maintaining this pathogen in the local transmission cycle. Although this study did not directly assess pathogen presence in host tissues, the consistent detection of *A. phagocytophilum* in ticks feeding on roe deer each year from 2018 to 2020 supports the possibility that *C. p. tianschanicus* contributes to the environmental persistence of this pathogen. Considering the high density of roe deer on Jeju Island and their frequent contact with tick vectors, livestock, and humans, further host-targeted studies, including molecular and serological analyses, are needed to evaluate their potential role as a reservoir host.

However, the vector competence of these ticks remains to be elucidated. *Ehrlichia* spp. was predominantly found in *H. flava*. Although sequencing was unsuccessful, the observed prevalence underscores the need for further molecular characterization of Ehrlichial agents in endemic ticks. *Babesia* spp. was detected in a single *H. megaspinosa* specimen, resulting in a minimum infection rate (MIR) of 20 %. Although this finding is consistent with previous reports from Japan (Andoh et al., 2014; Furuno et al., 2017; Yoshimoto et al., 2010), the small sample size (n = 5) and limited detection preclude any inference of vector competence. Given the possibility that the detected DNA may originate from residual host blood, further experimental studies are needed to assess the vector competence of *H. megaspinosa* for *Babesia* spp. Although feeding status was recorded as either fed or unfed for each tick, pooling was performed based on host individual and species, which may have resulted in mixed pools containing both fed and unfed specimens. Therefore, pathogen detection in some pooled samples may not directly indicate tick infection, and the possibility that some results reflect the presence of host blood cannot be excluded. This limitation should be considered when interpreting the prevalence of the pathogen. It should also be noted that sequencing was unsuccessful for both *Babesia* and *Ehrlichia* spp. Despite qPCR detection, the identification of these pathogens remains tentative. These limitations highlight the need for more robust molecular confirmation in future studies, potentially involving re-extraction, alternative primer design, or high-throughput sequencing approaches.

Phylogenetic analysis revealed that *H. megaspinosa* is genetically distinct yet closely related to *H. japonica*, underscoring the importance of combining morphological and molecular approaches in the identification of tick species.

This study has several limitations. First, the small number of *H. megaspinosa* specimens restricts generalization. Second, pathogen detection did not yield complete sequence data for *Babesia* and *Ehrlichia* spp., indicating the need for improved nucleic acid preservation or more comprehensive sequencing methods. Third, the study was geographically limited to Jeju Island, and the distribution of *H. megaspinosa* in the mainland remains unknown. Fourth, although ticks were stored at −70 °C, the delayed processing in 2023 raises the possibility that long-term storage may have contributed to nucleic acid degradation, particularly affecting the detection of labile RNA pathogens such as SFTSV. Given that SFTSV was not detected in any of the samples, this limitation may have contributed to false-negative results. More rigorous RNA preservation and earlier processing are needed in future studies involving RNA viruses. Fifth, ecological or habitat-based analysis was not possible due to missing or imprecise metadata; collection locations were often recorded only as “mountain,” preventing accurate association with environmental context.

This study provides updated data on the tick species infesting roe deer on Jeju Island, as well as the presence of three major tick-borne pathogens: *Anaplasma* spp., *Babesia* spp., and *Ehrlichia* spp. While *A*. *phagocytophilum* was confirmed through sequencing, the detections of *Babesia* and *Ehrlichia* require further molecular characterization. Notably, this is the first report of *H. megaspinosa* in the ROK, and the detection of *Babesia* spp. in this species—despite the small sample size—mirrors findings from Japan and raises questions about its potential vector role. Its close genetic relationship to Japanese populations and collection from inland areas suggests a possible introduction via migratory birds.

These findings underscore the importance of continued surveillance and vector competence studies to better understand the ecology of emerging ticks and tick-borne diseases, particularly on Jeju Island under shifting climatic conditions.

## CRediT authorship contribution statement

**Jong-Uk Jeong:** Writing – review & editing, Writing – original draft, Visualization, Methodology, Investigation, Formal analysis, Data curation, Conceptualization. **Hyun-Jeong Kim:** Supervision, Resources, Project administration, Funding acquisition. **Da-Seul Seong:** Methodology, Investigation, Formal analysis. **Hae-Eun Kang:** Supervision. **Jeong-Hee Han:** Supervision. **In-Soon Roh:** Supervision, Resources, Project administration, Methodology, Funding acquisition, Conceptualization.

## Ethical statement

This study was conducted under the ethical guidelines approved by the Institutional Animal Care and Use Committee (IACUC) of the Animal and Plant Quarantine Agency, Republic of Korea (ROK) (approval numbers: 2018–401, 2019–442, and 2020–514).

## Availability of data and materials

Data will be made available on request.

## Funding

This study was supported by a grant (N-1543085-2017-26-0108) from the 10.13039/501100008771Animal and Plant Quarantine Agency of the Republic of Korea.

## Declaration of competing interest

The authors declare the following financial interests/personal relationships which may be considered as potential competing interests:In-Soon Roh reports financial support was provided by 10.13039/501100008771Animal and Plant Quarantine Agency. If there are other authors, they declare that they have no known competing financial interests or personal relationships that could have appeared to influence the work reported in this paper.
